# Novel devices for intraoperative visualization in neurosurgical procedures: current state and prospect of using the exoscope

**DOI:** 10.1007/s00701-021-04839-7

**Published:** 2021-04-13

**Authors:** Joachim Oertel, Doerthe Keiner

**Affiliations:** Department of Neurosurgery, Saarland University Medical Center and Saarland University Faculty of Medicine, Kirrberger Str. 90.5, 66421 Homburg, Saar Germany

**Keywords:** Intraoperative exoscope, Operating microscope, Neuroendoscopy, HD-visualization

In the article ‘Beyond magnification and illumination: Preliminary clinical experience with the 4K 3D ORBEYE™ exoscope and a literature review’, Amoo and his colleagues introduce readers to their own experience with the ORBEYE™ exoscope (Olympus, Tokyo, Japan) in a series of 18 neurosurgical procedures. This device enables a high-resolution, three-dimensional (3D) magnified and illuminated intraoperative visualization of the surgical field on a 55-inch monitor using special 3D glasses. Contrary to ‘traditional’ endoscopic systems that provide two-dimensional (2D) images on the monitor, the exoscope might serve as an alternative to the ‘classic’ operating microscope (OM) with stereoscopic visual and illuminational characteristics. Thus, Amoo et al. focused on the aspects of intraoperative visualization and on the proposed possible ergonomic advances compared to the OM. Besides evaluation of their own experience, they reviewed the current literature of the use of different 4K 3D exoscopes in the field of neurosurgery.

The two following aspects are important: first, the technical development of the OM and, second, the technical development of the (neuro)endoscope since the beginning of the twentieth century.

Despite the remarkable technical development of neurosurgical armamentarium over the past decades including novel optic systems, so far, the OM is one of the most important tools. It enables ‘direct’ stereoscopic visualization. The ENT surgeon Carl Olof Nylén (1892–1978) was the first physician, who applied a (monocular) surgical microscope in the operation room at the University of Stockholm in 1921 [4, 5]. One year later, the University’s head of the department Gunnar Holmgren (1875–1954) used a binocular operating microscope attached with a light source [4, 5]. Although being used by an increasing number of ENT surgeons in the following years, it took more than 30 years to introduce the microscope into the neurosurgical operating room. In 1957, Theodore Kurze removed a neurilemmoma from cranial nerve VII in a 5-year-old patient [4, 8], and in 1965, the operative microscope was used for intracranial aneurysm surgery by the surgeons J. Lawrence Pool and Robert P. Colton [4, 9].

Today, neurosurgery is the leading field in using the operating microscope and its additional elements such as high-definition (HD) monitors, fluorescence imaging and image-guided surgical features [4].

Since the invention of Zeiss OPMI 1 in 1953, constant improvements regarding handling of the OM and technical applications were developed [4, 8]. Besides technical improvements for better illumination, ranges of magnification and longer working distance and changes concerning stability, flexibility, share of view and ergonomic and manoeuvrability were made. One important aspect is the share of view of the surgeon, the surgeon’s assistant and the scrub nurse by the attachment of a second eyepiece and a screen respectively. In general, modern OM are equipped with a selection of different binoculars for rotation in different heights and positions, full range of movement and several ranges of tilt of the optics carrier. Further, the newer generations’ OM has large HD monitors and tools for eye-to-object distance to prevent fatigue [4].

Although the handling of the surgical microscope has improved largely over the past decades, a comfortable and flexible working position for surgeons and assistants can still be challenging at times — especially in certain surgical procedures (i.e. procedures that require semi-sitting position) that can last several hours.

Besides the use of an OM, minimally invasive neuroendoscopic surgery is a major development of the past decades that has profoundly changed the neurosurgical approach in different intracranial and spinal pathologies. Today, in many centres, it is the preferred technique in pituitary adenoma resection, intraventricular tumour resection/biopsy and in the treatment of obstructive hydrocephalus. The technique has evolved since its beginnings in the early twentieth century resulting in the high-end neuroendoscopic armamentarium we use today. This is crucial especially for neurosurgical procedures. In today’s routine, high-definition camera systems, rigid rod-lens scopes with different angles and steerable scopes combined with frame-based or frameless neuronavigation are commonly used [2]. Like modern OM, ‘modern neuroendoscopy’ would not have been possible without the improvements in the optical quality of endoscopes and the basic research and inventions. Harold Horace Hopkins (1918–1994), a professor of physics at the University of Reading, developed the idea of using a bundle of glass fibres for image transmission over a long distance leading to a coherent image [2, 3]. For appropriate use in the medical field, the gastroenterologist Basil Hirschowitz continued to work on the technique and improved the original technique by using a different glass fibre material and a permanent coating that lead to a flexible fiberscope [2]. Eventually, the development of rigid rod-lens endoscopes combined with a cold light source and introduction of video cameras for imaging instead of direct observation by the surgeon looking through the endoscope enabled the application of this technique in the neurosurgical field. Since the end of the 1980s, image quality has continuously improved and thus could be adapted to different neurosurgical procedures.

Considering the recently launched exoscopes in the field of neurosurgery, dealing with the microscopic and endoscopic aspects is of importance. Very small and deep-seated anatomical structures and pathologies with close proximity to eloquent areas demand a high resolution, a sufficient magnification and a sufficient illumination. Dependent on the underlying pathology, the surgical microscope and the neuroendoscope meet these very important requirements for a safe and successful procedure.

In the recent publication of Amoo and colleagues, they used the ORBEYE™ exoscope in different intracranial pathologies including surgical resection for intracranial metastases, meningiomas, glioblastomas, a craniopharyngeoma, a radiation-induced necrosis, an arteriovenous malformation, a schwannoma and hemi-facial spasm. In 4/18 procedures, the approach was frontal interhemispheric (*n* = 1), retro-sigmoidal (*n* = 2) and sub-occipital (1×). In their series, they did not observe any complications related to the use of the exoscope. In all, the authors found the application to be feasible with a visualization that is at least comparable to the OM. Further, they stated that the angulation of the ORBEYE™ exoscope allowed a comfortable posturing of the surgeon especially in case of a retrosigmoid approach. Thus, one of the main advantages of the tool seems to be the ergonomic aspect.

pt?>In the passage ‘The exoscope in practice’, the authors compared their own experience with the ORBEYE™ to the experience of other study groups with available exoscopes of different companies such as VITOM by Karl Storz and KINEVO, a hybrid device of an OM and an exoscope, by Carl Zeiss. Contrary to the ORBEYE™, other investigators including the reviewer’s study group experienced issues with the depth of field, the illumination and ease of repositioning while using the VITOM-3D [1, 6]. In this context, it is of importance to notice that the issues were predominant in spinal procedures. Another study group evaluated the KINEVO 3D4K exoscope in a prospective-randomized clinical evaluation [7]. Although they observed a comfortable posturing, they had issues with the depth of field especially in case of deep-seated areas, too. They even switched from the exoscope to the OM in 50% of cases. The results indicate that the different model of exoscopes seem to be different in the intraoperative handling and in the quality of the digital image. Additionally, there might be differences in the handling and suitability of the devices for intracranial and spinal procedures.

In all, two characteristics of the exoscopes were observed that are promising. First, in most series, the ergonomic handling and the ease of intraoperative positioning of the device were found to be beneficial. Second, the ‘sharing of information’ that might even lead to an improved involvement of the co-surgeon and the scrub nurse during the procedure was found to be advantageous. While the authors of the present series were satisfied with the high-resolution 4K 3D digital images during surgery, other investigators were not satisfied with the visual quality. In the future, the experience of a larger number of surgeons in different neurosurgical procedures will show if the exoscopes are a valuable addition or can even serve as an equivalent replacement of the OM.

However, the evolution of the operative microscope and the endoscope from the early twentieth century to date demonstrated the importance of a constant effort to improve the available techniques. Every surgeon — or ‘user’ of these devices — should be curious about new developments, because they might add value in the best neurosurgical treatment and patient care possible.

## Abbreviations

2D: Two-dimensional
3D: Three-dimensional
OM: Operating microscope
ENT: Ear nose throat
HD: High definition
